# Supplementary figures and images for: Evaluation of the reliability and risks of ChatGPT-4o in answering pediatric cough questions: A comparative analysis between pediatricians and pediatric pulmonologists
Source: PLoS One. 2025 Dec 31;20(12):e0340007. doi: 10.1371/journal.pone.0340007 (PMC12755730; doi:10.1371/journal.pone.0340007)

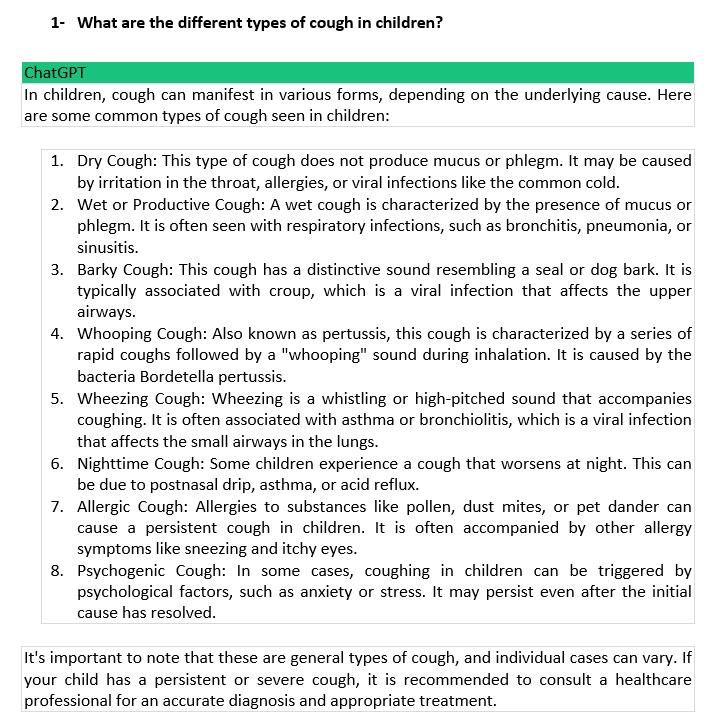


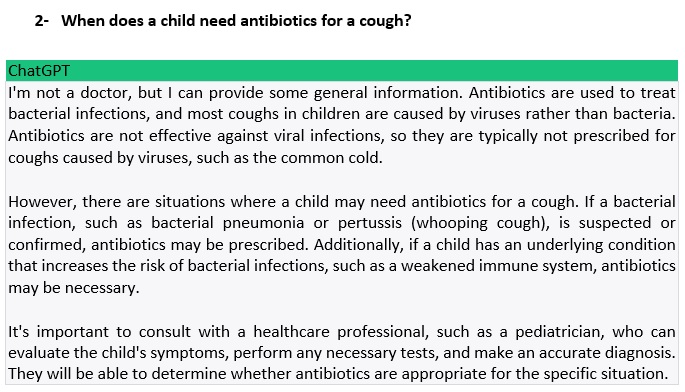


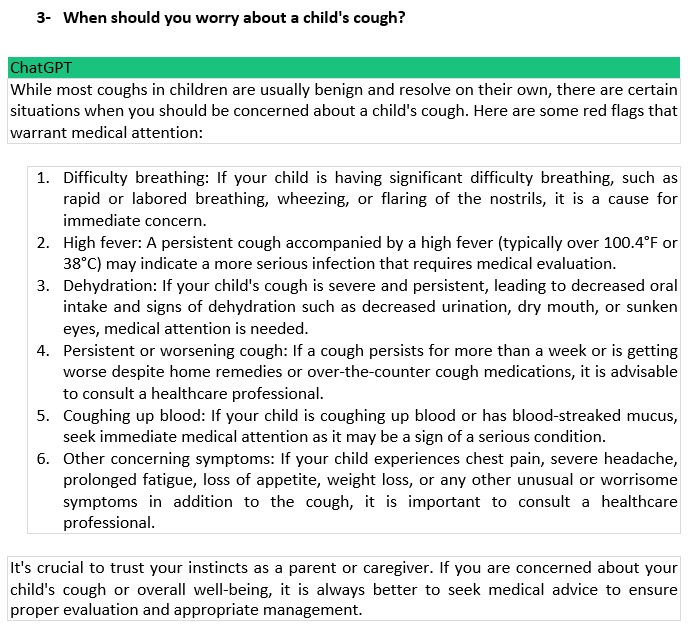


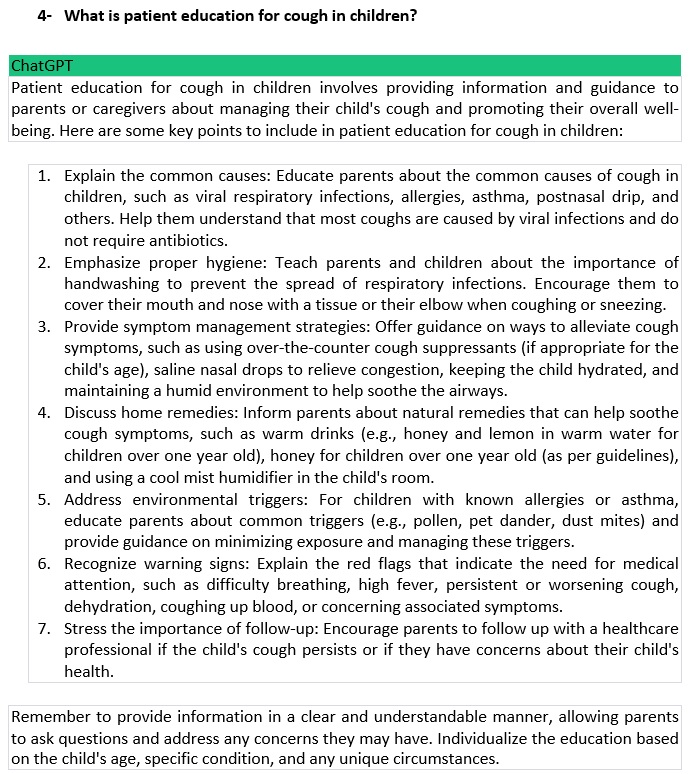


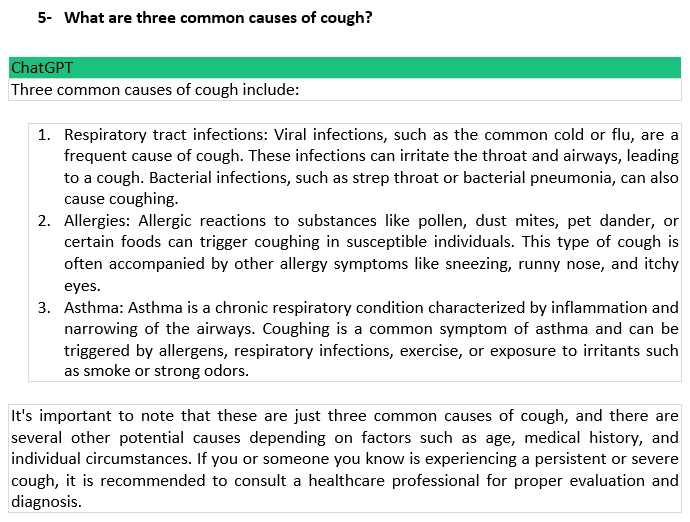


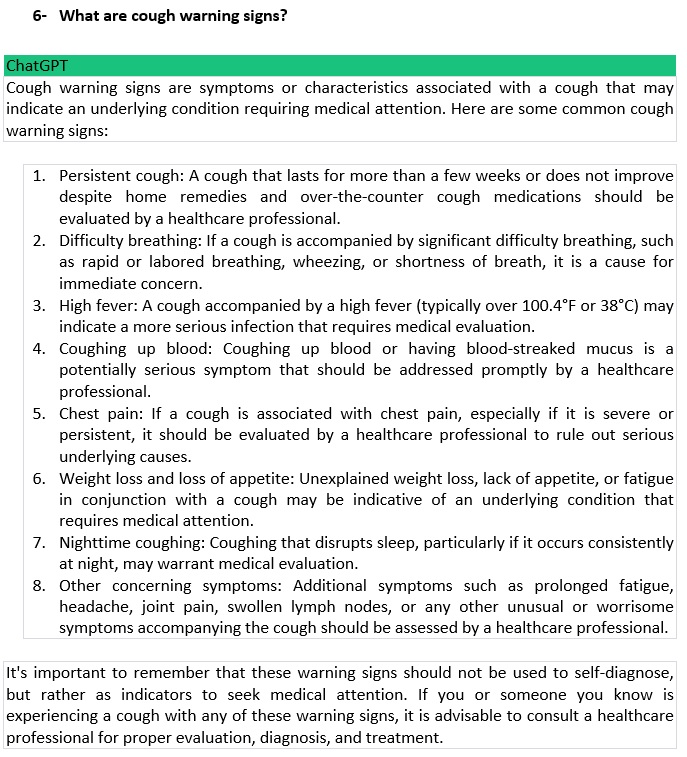


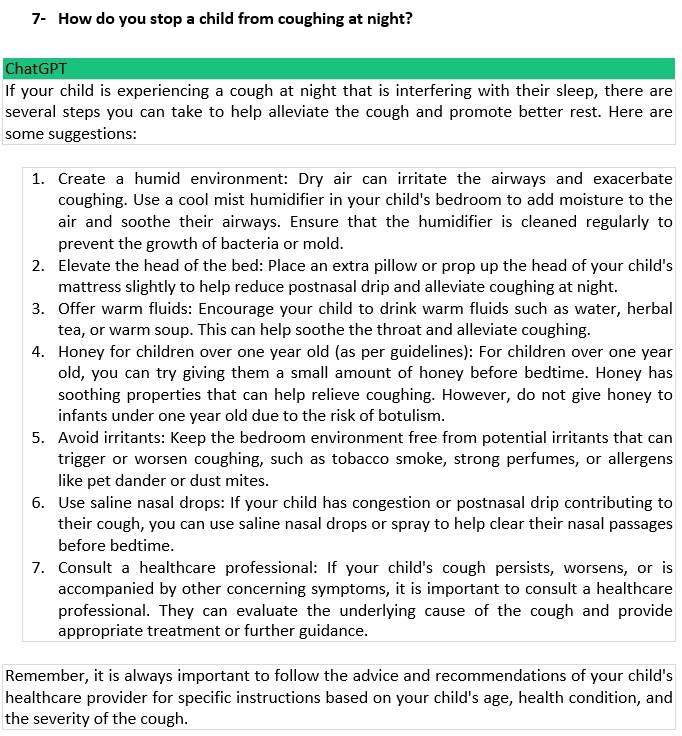


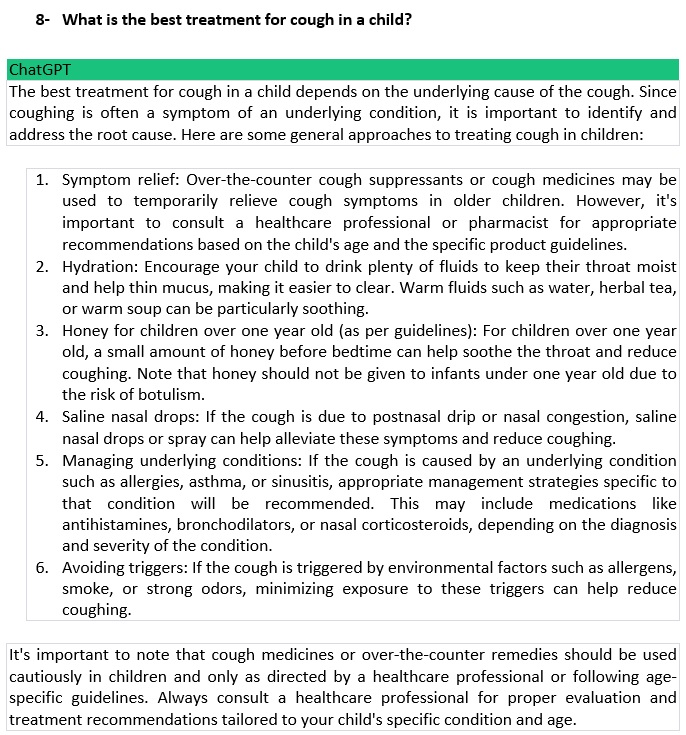


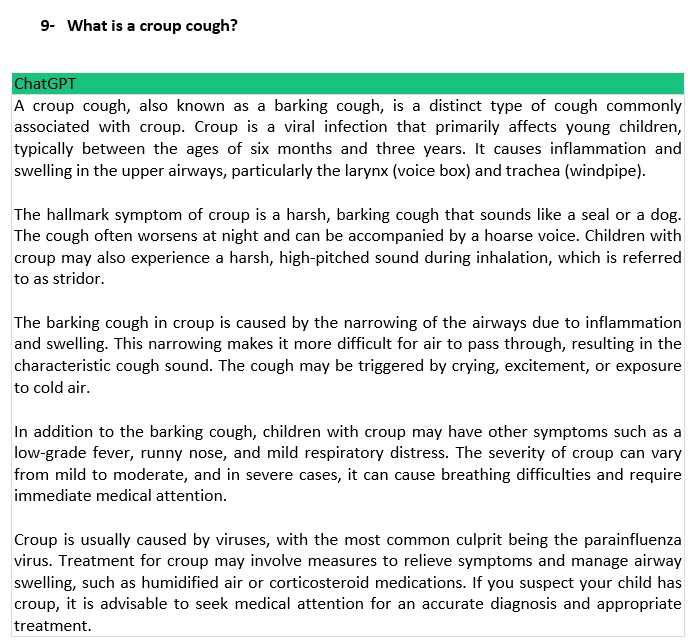


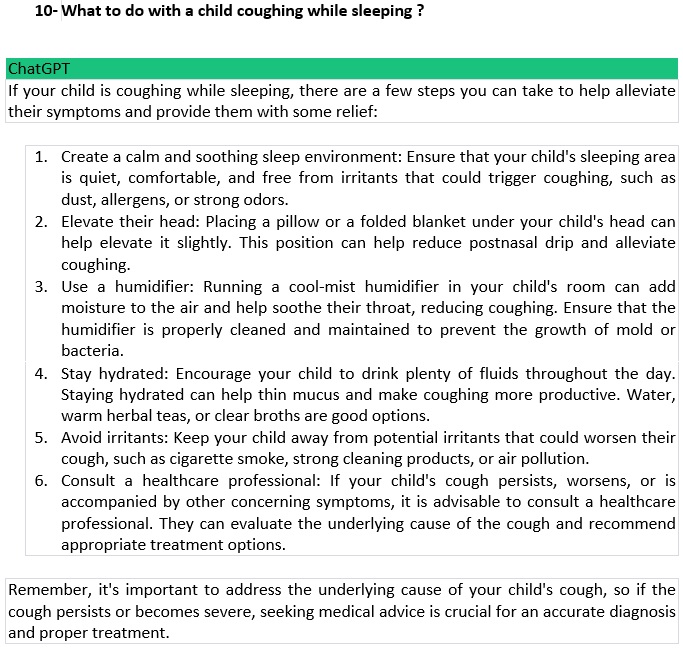

Supplement: S1 File — (DOCX) [file pone.0340007.s001.docx]
