# Supplementary material for: Evaluation of the reliability and risks of ChatGPT-4o in answering pediatric cough questions: A comparative analysis between pediatricians and pediatric pulmonologists
Source: PLoS One. 2025 Dec 31;20(12):e0340007. doi: 10.1371/journal.pone.0340007 (PMC12755730; doi:10.1371/journal.pone.0340007)
Supplement: Attachment — Submitted filename: Response to reviewersR1.docx [file pone.0340007.s004.docx]

**Dear Editor and Reviewers,**

We sincerely thank you for the thoughtful and constructive feedback provided on our manuscript titled **“Evaluation of the reliability and risks of ChatGPT-4o in answering pediatric cough questions: a comparative analysis between pediatricians and pediatric pulmonologists.”** We carefully considered each comment and revised the manuscript accordingly. Below we provide a point-by-point response, detailing how each concern was addressed.

We believe these revisions have substantially strengthened the clarity, methodological rigor, and interpretability of our study.

**EDITOR COMMENTS**

**Editor Comment 1**

*“Study design lacks systematic methodology and validation. The design does not include open-ended questions which can help understand the limitations of ChatGPT responses.”*

**Response:**
We agree with the editor’s concern. We added a justification for not including open-ended items and acknowledged this as a study limitation. Specifically:

- **Methods → Study Design:** We explained that open-ended questions were considered but excluded to minimize participant burden and maintain response rates in a geographically dispersed physician sample.
- **Discussion → Limitations:** We now explicitly state that the absence of qualitative explanations limits interpretation and that future studies should incorporate open-ended follow-up questions.

**Editor Comment 2**

*“Authors should discuss why ChatGPT was selected for this study when several AI models exist.”*

**Response:**
A new paragraph was added in the Methods section explaining why ChatGPT-4o was selected, emphasizing its widespread use, public accessibility, multilingual capacity, and performance in recent medical evaluation studies.

**Editor Comment 3**

*“Statistical analysis: The study is underpowered to detect small differences. Suggest adding effect sizes for all comparisons; report power analysis; include 95% CIs; include boxplots for Likert ratings.”*

**Response:**
All recommended improvements were implemented:

- **Effect sizes (r) and 95% confidence intervals** were added for all Mann–Whitney U analyses (Tables 1 and 2).
- A **post hoc power analysis** using G*Power 3.1 was conducted and reported in the Statistical Analysis section.
- **Boxplots** illustrating the distribution of Likert ratings for both specialties were added as **Figure 1**.

**Editor Comment 4**

*“Discussion: Discuss pulmonologists’ responses and whether associated with case complexity; discuss study limitations such as subjective Likert ratings.”*

**Response:**
We expanded the Discussion to include:

- A detailed explanation of why pediatric pulmonologists provided more critical ratings (complex cases, rare presentations, greater sensitivity to nuance).
- An explicit acknowledgment of Likert-scale subjectivity and the absence of formal psychometric validation.
- An addition on content/face validity established through expert review.

**REVIEWER COMMENTS**

**Reviewer Comment 1**

*“An introduction to the abstract is not written.”*

**Response:**
We revised the abstract to include a clear introductory sentence that establishes the rationale behind the study.

**Reviewer Comment 2**

*“What was the rationale for specifically using the 10 questions presented?”*

**Response:**
We added a statement explaining that the selected questions consistently appeared across repeated unbiased Google searches and represent the most common parent-driven concerns in pediatric respiratory practice.

**Reviewer Comment 3**

*“The sample size calculation is not mentioned.”*

**Response:**
A **post hoc power analysis** was conducted and added as recommended, showing adequate power (>80%) for the observed effect sizes.

**Reviewer Comment 4**

*“The Likert scale was not tested for reliability and validity.”*

**Response:**
We now provide a detailed methodological explanation:

- The four Likert items intentionally measured distinct constructs, so internal consistency metrics (e.g., Cronbach’s alpha) were not appropriate.
- However, content and face validity were ensured through expert review by two pediatric pulmonologists.
- The absence of formal psychometric validation is explicitly discussed as a study limitation.

**Reviewer Comment 5**

*“Adding the reasons behind ‘yes’ answers would have given better context.”*

**Response:**
We fully agree. We explicitly acknowledged this as a limitation and clarified that lack of qualitative explanations restricts interpretability. We also recommended including open-ended follow-ups in future research.

We thank the editor and reviewers for their valuable feedback. We believe the revisions have substantially improved the quality, methodological transparency, and clarity of the manuscript. We hope the revised version meets your expectations and look forward to your favorable consideration.

**Sincerely,
The Authors**
